# Multiscale Characterization of Electrode-Induced Degradation in Perovskite Solar Cells

**DOI:** 10.1021/acsaem.5c03347

**Published:** 2026-02-16

**Authors:** Goutam Paul, Jackson W. Schall, Harvey L. Guthrey, Marc Migliozzi, Robert Tirawat, Dennice M. Roberts, Steven W. Johnston, Mowafak M. Al-Jassim, Chun-Sheng Jiang, Axel F. Palmstrom, Dana B. Kern

**Affiliations:** 53405 National Renewable Energy Laboratory, 15013 Denver West Parkway, Golden, Colorado 8040, United States

**Keywords:** perovskite solar cells, dark storage degradation, electrode corrosion, shelf life, electroluminescence and photoluminescence imaging, scanning electron microscopy, Kelvin probe force microscopy

## Abstract

The stability of metal-halide-perovskite (MHP) solar cells must be understood and improved for the commercial viability of MHP technologies. Here, we apply multiscale characterization methods to study degradation modes, specifically electrode corrosion, for p-i-n MHP partial device stacks and full devices that are stored in the dark under an inert atmosphere. Our multiscale characterization approaches include full-device electro-optical performance using current–voltage (JV) curves and spatial imaging with electroluminescence (EL) and photoluminescence (PL). We further correlate interface properties using cross-sectional Kelvin probe force microscopy, which maps the nanoscale electric field properties, and electron microscopy, which demonstrates structural and chemical features. Devices stored as a full device stack degrade primarily by metal (Ag) electrode diffusion into the absorber, with formation of AgI byproducts and Ag accumulation near the indium tin oxide (ITO) contact. This causes decomposition of the perovskite absorber domains, loss of the potential drop at the electron transport layer (ETL)/perovskite interface near the metal contact, and increased equivalent resistance at the perovskite/hole transport layer (HTL) interface near the ITO contact. The devices stored without metal show a different degradation pathway dominated by corrosion of the ITO, creating voids at the ITO electrode surface with diffusion of In and Sn into the absorber. We conclude that metal electrode-induced degradation is the most severe degradation pathway under dark storage, but that ITO corrosion and absorber instability must also be mitigated. We further demonstrate mitigation of these degradation pathways by changes to the device stack, including a SnO_*x*_ blocking layer at the ETL side and replacing ITO with FTO at the HTL side. These results provide a useful demonstration of specific dark degradation pathways at each electrode interface, as well as a unique multiscale example that links degradation of chemical, structural, and electrical interface properties to the full-device electro-optical characteristics.

## Graphical abstract

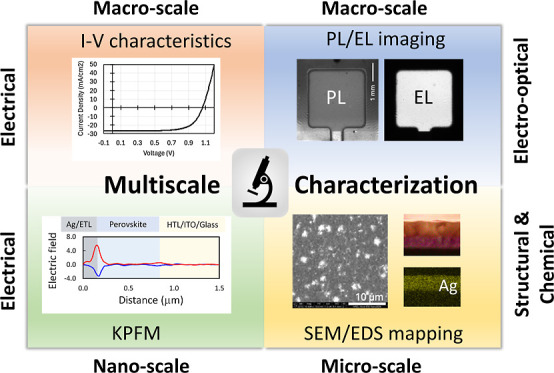

## 1 Introduction

The impressive improvement in metal-halide perovskite (MHP) solar cell efficiency from 3.8% to over 27.3% in the past 15 years has generated interest from both the research community and the photovoltaics industry.^1–5^ However, durability is still lacking, and a multitude of degradation modes must be understood and mitigated.^6^ A recent consensus on assessing perovskite device stability outlines the importance of evaluating degradation pathways under various controlled conditions, including dark storage, light soaking/cycling, voltage bias, elevated temperatures, and outdoor aging.^7^ The Perovskite Database Project^8^ shows that degradation under dark storage is the most widely investigated condition.^9^ Indeed, dark storage is an unavoidable baseline for durability testing, especially as it becomes more relevant during the shipping and installation of commercialized PV modules. Thus, understanding the intrinsic stability of PV components during dark storage is key to separating shelf life degradation from stress-induced effects and is important for understanding encapsulated vs edge-seal designs of modules.^10^

Degradation in the dark can proceed via various mechanisms. Decomposition of MHP absorbers may be activated in the dark even at low temperatures due to the materials’ low formation energies that enable low-temperature processing.^11^ Absorber decomposition generates byproducts such as volatile organics, amines, halide gases, PbI_2_, and Pb^0^.^12–14^ This both degrades the MHP semiconductor and induces reaction pathways with interface layers.^15^ Chemical degradation at interfaces may also proceed by dopants diffusing between the charge transport layers and absorbers.^16^ Built-in energetic and chemical gradients in full device stacks can further cause electrode-induced degradation in the dark by ion movement and associated reaction pathways.^17,18^ Ion migration, causing reactions between metal electrodes and halides, may result in electrode corrosion, especially if blocking layers are not used or degrade.^19–21^ Although contacts with transparent conductive oxides and metal oxide transport layers are presumed to be more stable than metal electrodes and organic transport layers, these interfaces may still degrade by electrochemical redox mechanisms induced by interfacial defects, ion migration, or reactions with the atmosphere.^22–25^

Many of the dark-storage degradation modes described above originate at or impact an interface between an electrode and the absorber. However, it can be difficult to identify which interface is limiting device stability or to separate the different degradation pathways at each interface. In particular, interface characteristics are not directly evident from common full-device electro-optical measurements that are frequently used to rapidly screen devices, such as current density–voltage (JV) curves and electroluminescence (EL)/photoluminescence (PL) characterization. Furthermore, most studies for interfacial root cause analysis utilize chemical and structural microscopy with depth profiling or cross-sectioning.^13,20,26–28^ Although such chemical analysis is very valuable, these compositional observations do not directly probe the nanoscale interfacial electrical characteristics that drive the device performance.

Here, we link chemical, structural, and electrical nanoscale interface characteristics for a comprehensive analysis of interface degradation during dark storage, as recommended in ISOS-D-1.^7^ We separate the degradation pathways originating at each electrode interface by comparing devices stored with metal electrodes or as partial device stacks with no metal for 1 year in an argon atmosphere. We demonstrate that different interfacial degradation pathways proceed depending on whether metal is present during dark storage. We put these interface degradation pathways in context with the full-device electro-optical performance. Our results provide an example of degradation mechanisms under dark storage, which must be understood as a baseline for MHP solar cell durability as this technology approaches commercial prospects.

## 2 Experimental Section

The perovskite devices in this study have a p-i-n construction including glass/indium tin oxide (ITO)/NiO_*x*_/N4,N4′-Di­(naphthalen-1-yl)-N4,N4′-bis­(4-vinylphenyl)­biphenyl-4,4′-Diamin (VNPB)/FA_0.87_Cs_0.13_Pb­(I_0.95_Br_0.05_)_3_/C_60_/bathocuproine (BCP)/Ag and an active area of 0.12 cm^2^. All substrates of this device construction, as well as substrates with all layers except the Ag electrode, were fabricated at the same time. Devices were characterized by current density–voltage (JV) curves and the imaging of photoluminescence (PL) and electroluminescence (EL). The devices, along with corresponding partial device stacks, were then stored in an argon glovebox within a glass jar wrapped in foil for 1 year. We characterized 36 devices in fresh condition, 6 devices after dark storage with metal, and 18 devices after dark storage with a new metal.

The JV curves were collected using a steady-state SunBrick LED solar simulator and a Keithley sourcemeter with a scan rate of 100 mV/s. No shadow mask was used during JV characterization, which may result in the overestimation of performance. We calculated the *J*_SC_ using the area of the metal pad, which was characterized by an optical microscope. PL and EL images were collected using a Princeton Instruments PIXIS Silicon CCD camera with a 715 nm long pass filter. PL was excited by 450 nm diffuse light at an equivalent of 1 Sun (100 mW/cm^2^) intensity from a high-power Prizmatix LED. EL was excited by current injection in forward bias, using the same current for all samples, equivalent to 0.1× the short circuit current taken from the nondegraded condition. Exposure times were 5 ms for PL and 0.5 s for EL.

Scanning electron microscopy (SEM) images and energy-dispersive X-ray spectroscopy (EDS) were collected on an FEI Nova NanoSEM operating with an accelerating voltage of 10 kV and incident beam current of ∼1.3 nA. An Oxford Ultimax EDS detector was used to produce elemental maps. The metal from the top surface of the device was removed using scotch tape to avoid the possibility of electron-beam-induced metal migration. The tape was gently pressed onto the silver electrodes and then peeled off, effectively lifting the metal because the silver electrode is poorly adhered to the weak fullerene electron transport layer (ETL) of the device.^10,29^

For both SEM and Kelvin probe force microscopy (KPFM), devices were mechanically cleaved inside an Ar glovebox, and no additional polishing was performed to avoid modification of the cross-sectional surface. KPFM measurements used a Bruker D5000 atomic force microscope (AFM) with a Pt–Ir coated silicon probe (Nanosensor PPP-EFM). The system has approximately 30 nm spatial resolution and a 10 mV electrical resolution. Topography and potential maps were obtained at the same time in tapping mode. External electrical bias was applied between the ITO and Ag electrodes using a Keithley sourcemeter. To obtain the electric field profiles induced by the external bias voltage, we first extracted potential profiles by averaging line scans from the potential images acquired under each applied bias over several hundred nanometers. Potential difference profiles were then calculated by subtracting the 0 V potential profile from those obtained at different bias voltages (*V*_*b*_). The electric field profiles were subsequently determined by taking the first derivative of the potential difference profiles.

## 3 Results and Discussion

### 3.1 Electro-Optical Parameters

To investigate degradation mechanisms under dark storage, we aged p-i-n MHP samples in an argon glovebox for 1 year, including full devices with metal electrodes (ITO/NiO_*x*_/VNPB/MHP/C_60_/BCP/Ag) along with samples fabricated at the same time that did not have a metal electrode (ITO/NiO_*x*_/VNPB/MHP/C_60_/BCP). Figure 1 shows PL images, EL images, and JV curves for these devices, including: (a) a representative device immediately after fabrication, (b) the same device again after 1 year of dark storage, and (c) a partial device stack fabricated at the same time that was stored without the metal electrode but which had a fresh metal electrode applied for characterization.

**Figure 1:**
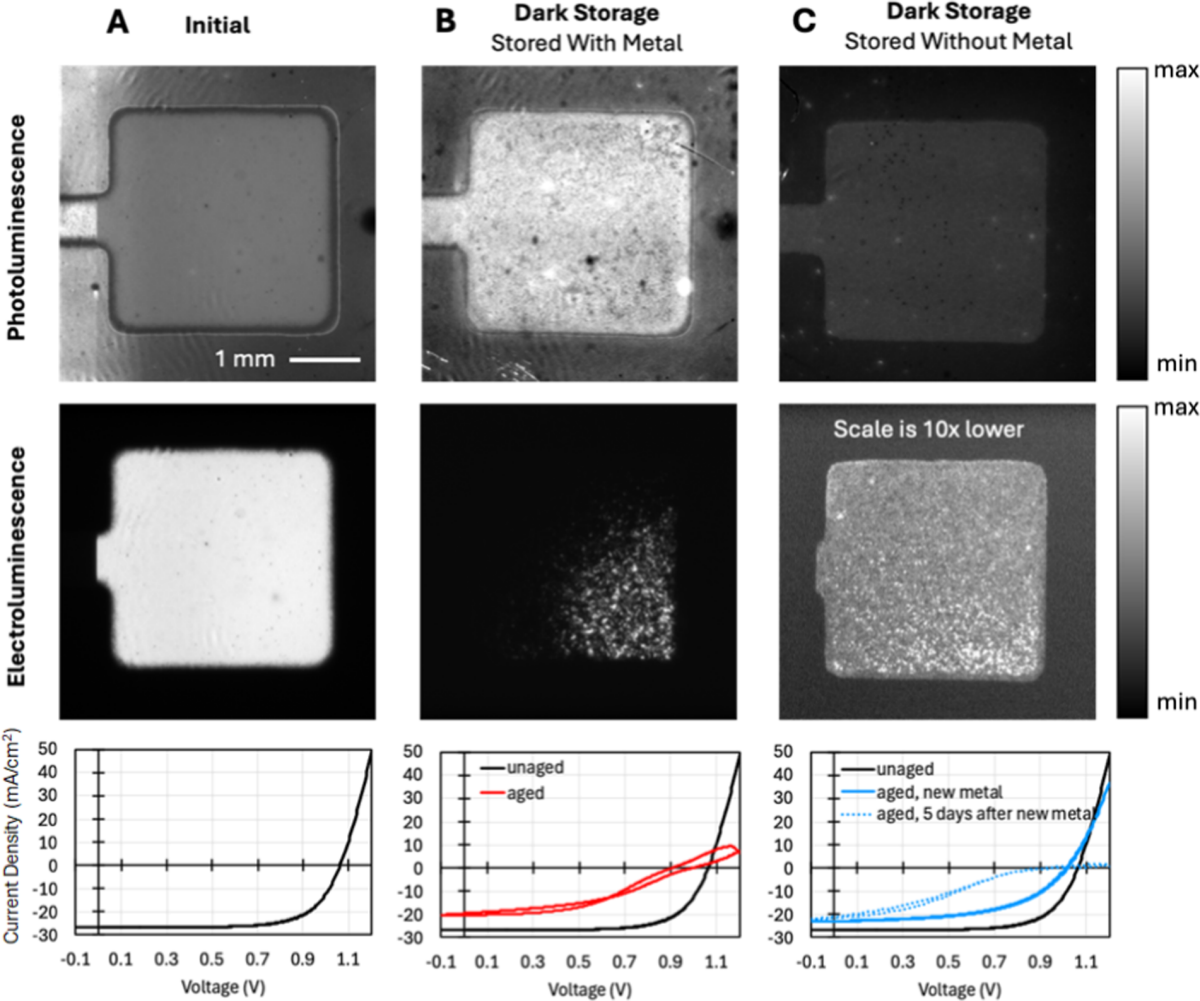
Comparison of photoluminescence images, electroluminescence images, and current–voltage curves for the same device (a) before and (b) after 1 year dark storage, along with (c) a partial device stack fabricated at the same time as (a,b) that was stored without metal and had metal applied for characterization after 1 year of dark storage. EL/PL images were recorded after 5 days after new metal deposition.

The device immediately after fabrication shows a uniform PL and EL signal (Figure 1a). After one year of dark storage (Figure 1b), the EL image becomes dark in most of the device area, with a speckled “starry night” pattern in part of the device. This EL pattern suggests significant electrical contact degradation, resulting in only a few isolated areas where current can flow into the device. That is, much of the electrical contact has become highly resistive, and the speckled area represents a few low-resistance areas where current crowding generates a high EL signal.

The PL image of Figure 1b shows approximately 2× higher intensity after dark storage, along with increased nonuniformity. The increase in PL intensity may arise from degradation of the charge extraction layers,^30^ absorber degradation that generates emissive defects or byproducts,^31,32^ or light scattering from the electrode degradation. We note that the speckled areas that are bright in the EL appear darker in the PL image (Figure 1b, top). This may imply these speckles are either local shunts or small areas where charge extraction layers maintain low series resistance. The associated JV curve shows a loss of current density (*J*_SC_) as well as the development of hysteresis and a severe decrease in the fill factor (FF). The change in slope of the JV curve suggests both increased series resistance as well as decreased shunt resistance.

These trends are indicative of interfacial degradation and possible bulk film degradation. Increased series resistance often arises from the formation of insulating interface layers due to metal diffusion, ion migration, or decomposition of charge transport layers. Decreased shunt resistance suggests the presence of new leakage pathways. Bulk degradation of the MHP active layer may simultaneously occur due to diffusion of metal ions. Collectively, these factors contribute to severe charge extraction losses and an increased level of recombination. Figure S1 of the Supporting Information gives further insight into these degradation pathways with X-ray diffraction (XRD) data identifying degradation products of AgI, PbI_2_, and an additional perovskite phase in this aged stack.

The device fabricated from a partial stack that was aged without metal (Figure 1c) shows a different degradation behavior compared to the device that was aged with metal. Immediately after applying fresh metal to the aged partial device stack, the JV curve shows a relatively small amount of degradation. While *J*_SC_ losses appear similar to those of the device aged with metal, the FF loss is not as severe. This condition aged on only ITO with freshly deposited metal reveals the relative severity of metal-induced degradation (Figure 1b) compared with degradation dominated by the absorber on an ITO contact (Figure 1c): Metal-dominated degradation resulted in 58 ± 8% loss in power conversion efficiency, and degradation without the metal resulted in degradation of only 37 ± 5%.

However, remeasuring this device after 5 additional days of dark storage demonstrates instability. That is, we observe continued FF loss and development of an injection barrier indicated by the roll-over near the open-circuit voltage (*V*_OC_).^33^ We propose that the continued degradation of the JV curve over these 5 days after application of the fresh metal is due to corrosion of the metal contact. It appears that the aged absorber/C_60_/BCP surface may rapidly react with the Ag electrode. Figure S1 of the Supporting Information shows XRD data indicating PbI_2_ formation prior to metal deposition. This shows that the absorber degrades and halide-rich degradation products could react with the newly deposited Ag.^34,35^

Figure 1c also shows PL and EL images for the aged partial stack that received a fresh metal contact, measured after an additional 5 days of aging, which caused metal corrosion. We find that the PL intensity is about half of that of the unaged device from Figure 1a and 1 of the PL intensity from the device aged with metal from Figure 1b. This result demonstrates that the MHP layer has degraded under dark storage, resulting in nonradiative recombination. We note that the lower PL from increased nonradiative recombination is consistent with the decrease in *V*_OC_ observed for this device prior to the formation of the injection barrier, where small changes in *V*_OC_ cause large drops in PL due to their exponential relationship.^36^ It appears that the generation of highly emissive byproducts or light scattering (as observed in Figure 1b) occurs only when the full stack is aged in contact with the Ag metal.

The EL image of the device stored without metal in Figure 1c shows an intensity 10× lower than the unaged device, consistent with the development of a resistive injection barrier in conjunction with high nonradiative recombination in the MHP. While the EL for both aged device conditions shows some extent of the speckled starry night pattern, we note that the weak EL signal in the device aged without metal is more uniform over the device area compared to the device aged with metal in Figure 1b.

### 3.2 Interface Structural and Chemical Characterization

We next investigated the chemical and structural characteristics of the device layers after dark aging. Figure 2 shows cross-sectional SEM for several areas under each condition. Prior to cleaving, the metal electrodes were removed using scotch tape. We removed the metal to avoid possibilities of electron-beam-induced metal migration. The devices were then mechanically cleaved by scoring the glass side, and no additional surface preparation was used in order to avoid changing the microstructure.

**Figure 2:**
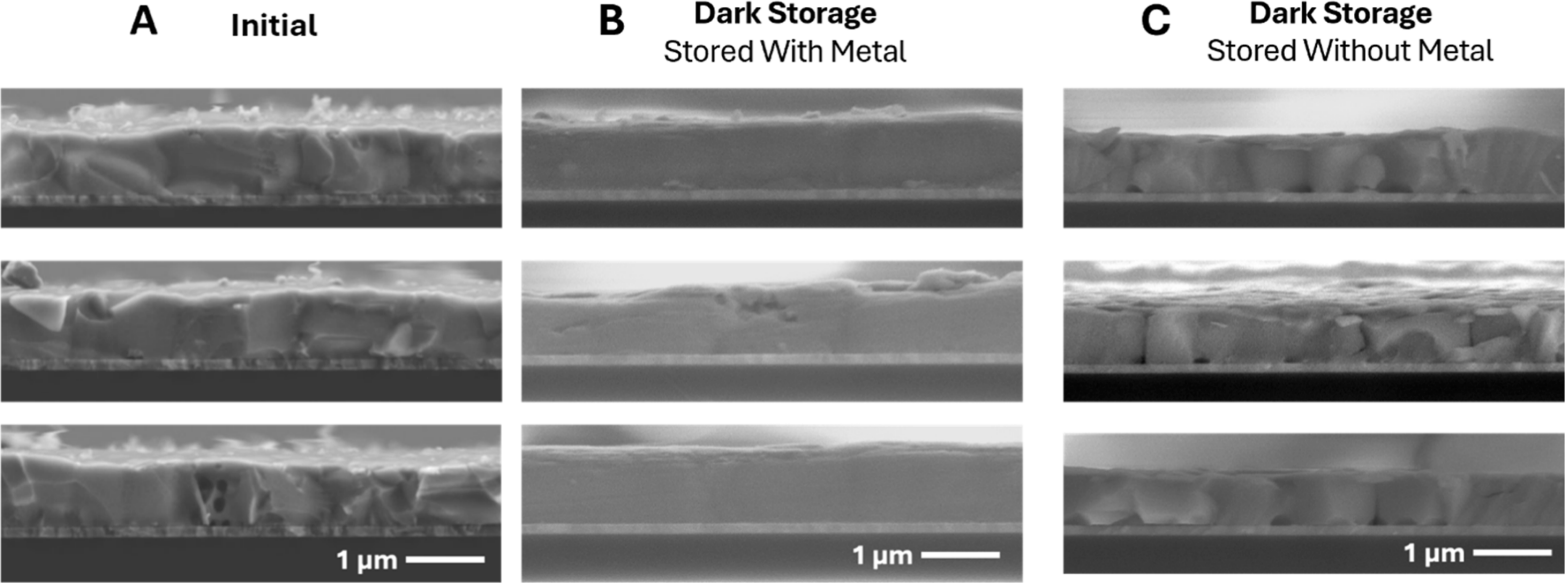
Cross-sectional SEM images from three different areas of each sample type, after Ag electrode removal, including (a) unaged control devices (b) devices stored in the dark for 1 year as a full device stack with metal, and (c) samples stored as a partial device stack with no metal for 1 year that had fresh metal applied for other characterizations (SEM images were recorded after >5 additional days of new metal deposition).

The cross-section of the unaged device (Figure 2a) shows a roughness corresponding to perovskite grain/domain boundaries. A few voids are observed in the absorber even in the unaged condition. Figure 2b shows a device that was stored with a metal. In this case, we observe a change in the perovskite morphology leading to a smooth absorber layer with no detectable domain boundaries, suggesting significant changes within the active layer. The XRD of fresh and degraded samples (Figure S1) shows the presence of Ag, AgI, more PbI_2_, and a possible additional perovskite phase in degraded samples after electrode removal, indicating the decomposition of the perovskite active layer and reactions with Ag.

Figure 2c shows the device stored without a metal contact. Here, the perovskite domain boundaries are still detectable. However, the lower PL intensity in Figure 1c and XRD results in Figure S1 indicate absorber degradation despite a smaller change in the morphology, suggesting a different degradation behavior of the absorber compared to the device stored with the metal electrode. Additionally, this device shows more voids at the ITO/HTL/MHP interface. The voids could form due to reactions at the HTL interface.^22,25,37^

We further investigated the chemical nature of the perovskite bulk and interfaces for the devices aged in dark storage. The top panel of Figure 3 shows the surface of the aged samples after the Ag metal electrode was removed with scotch tape. The sample aged as a full device with metal shows regions of bright contrast along the surface, corresponding to clusters of the remaining Ag electrode that could not be removed. This suggests that Ag became strongly adhered and may have migrated into the absorber during dark storage. The sample that was aged without metal shows a surface clear of Ag clusters. We note that the sample aged without metal had fresh Ag applied for JV, EL, XRD, and KPFM measurements, and the Ag was then removed with scotch tape after >5 additional days of dark storage.

**Figure 3:**
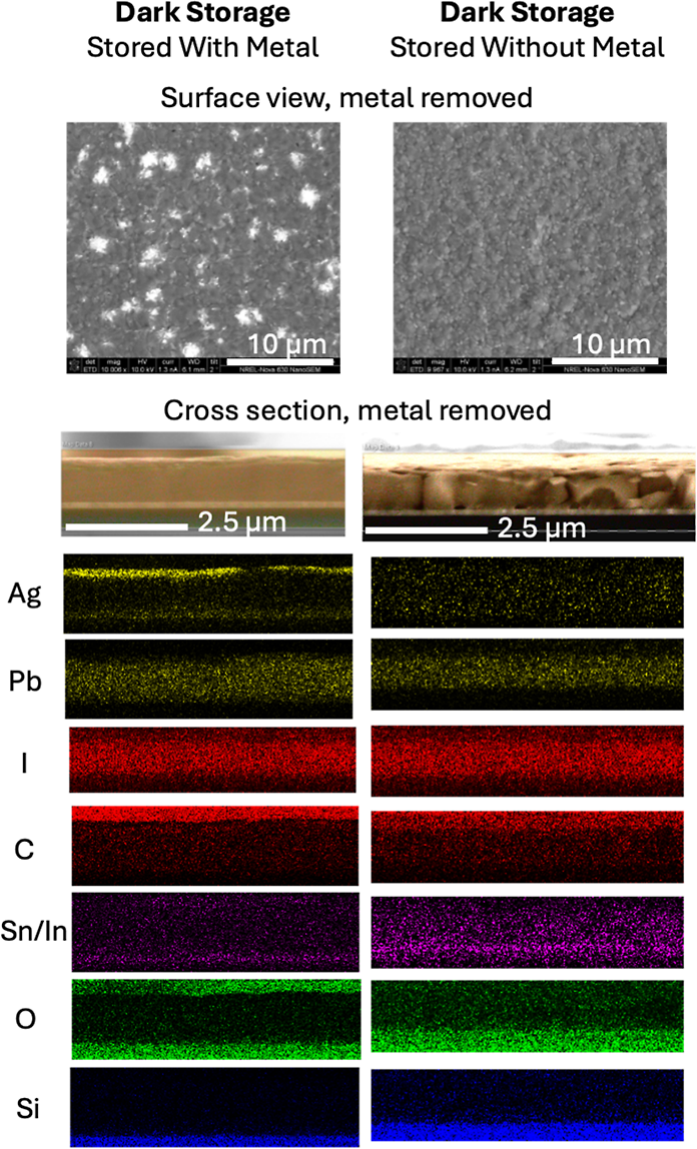
Comparison of degraded devices stored either as a full stack with metal (left) or as a partial stack without metal (right), including SEM surface view (top) or cross sections with elemental profiles by energy-dispersive X-ray spectroscopy (bottom).

The bottom panel of Figure 3 shows the cross-sectional energy-dispersive X-ray spectroscopy (EDS) profiles for these aged devices. The device aged with metal shows that Ag is still attached to the surface and could not be fully removed with scotch tape. We also observe that Ag has migrated into the absorber, and there is Ag accumulation near the ITO contact. The device stored without metal does not show significant Ag migration into the absorber. Taken together with the results of JV, PL and EL imaging, XRD, and KPFM, this result suggests that Ag migration after 1 year of dark storage with electrode attached causes shunting through the ETL side and detrimental chemical reactions within the absorber and at the HTL side.

Interestingly, the device stored without metal shows a broader Sn/In signal near the ITO contact extending into the MHP. We present the Sn and In signals together, as the dispersion used for EDS acquisition resulted in a 10 eV/channel energy resolution. This means that several of the L X-ray lines for Sn and In are separated by 5 or fewer channels, making them difficult to deconvolute within the broader EDS peaks for these elements that exhibit full-width-half-max values > 100 eV. Prior studies suggest that Sn and In ions may migrate into the perovskite layer due to perovskite degradation products that etch/corrode the ITO.^25,37^ The ITO corrosion reactions may generate voids in the absorber,^37^ consistent with our observation of a greater extent of voids near the ITO in Figure 2c. We note that Figure 3 further implies that Sn and In diffusion from the ITO into the absorber occurred to a greater extent when the partial device stack was stored without metal. We propose that the device stored with metal experiences a lesser extent of Sn or In diffusion from the ITO due to Ag migration and accumulation that indirectly impact the chemical reaction pathway at the ITO contact.

### 3.3 Interface Electric Field Characterization

Figure 4 shows external-voltage-induced electric field profiles of the same devices from PL/EL imaging and the SEM/EDS analysis discussed above. The electric field data are produced by applying external bias voltages of −0.5 V, 0 V, and +1 V between the ITO and metal contacts and using an AFM tip to map the cross-sectional surface potential. We subtract the 0 V surface potential from each biased measurement (−0.5 V and +1 V) to remove artifacts of static surface charge. The electric field profiles in Figure 4 are given as the derivative of the subtracted surface potential profiles.

**Figure 4:**
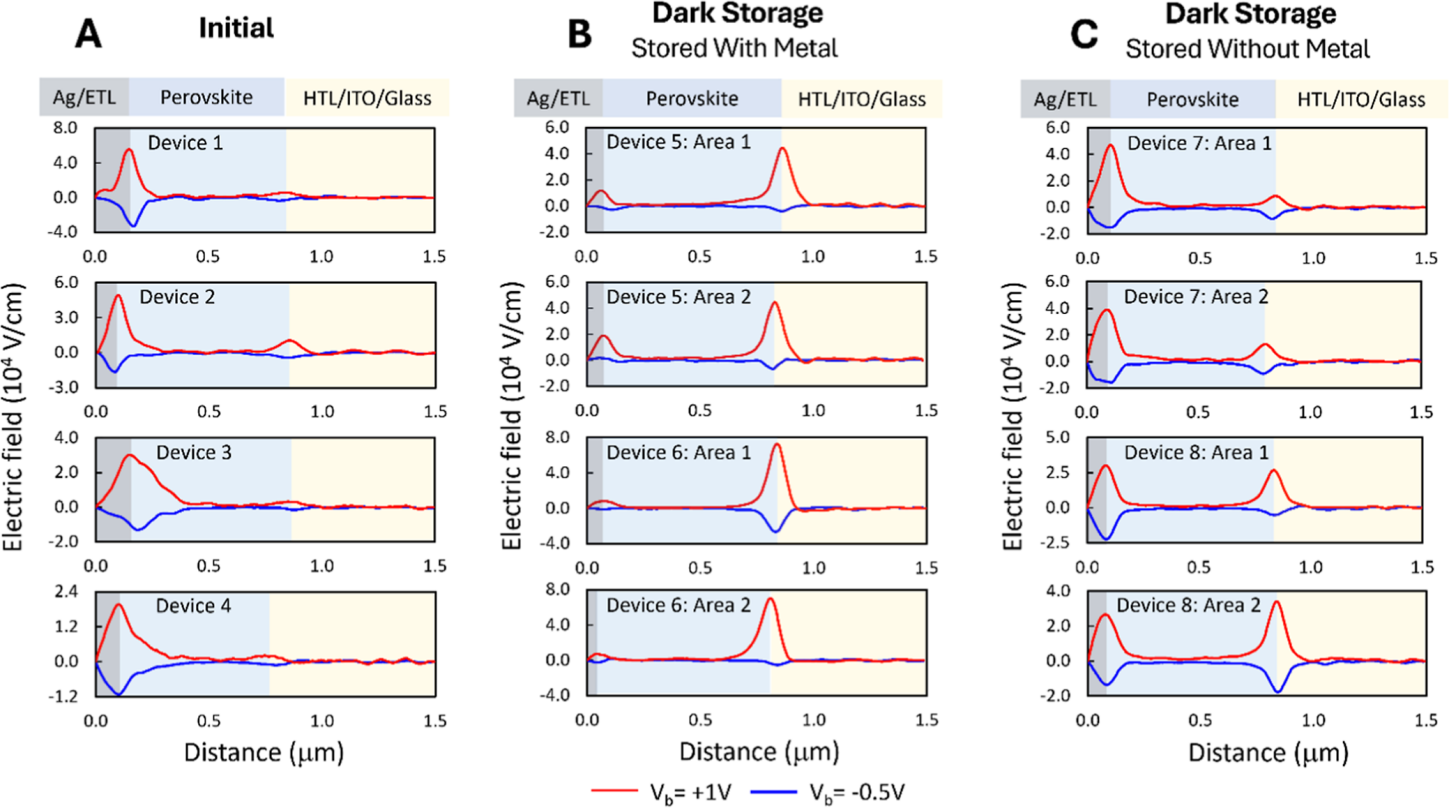
Electric field profiles from cross-sectional KPFM measurements representing (a) unaged control devices, (b) devices stored in the dark for 1 year as a full device stack with metal, and (c) samples stored as a partial device stack with no metal for 1 year, with fresh metal applied for characterization (KPFM was measured after >5 additional days of new metal deposition).

The KPFM measurements provide electric field peaks that relate to potential drops caused by resistance to current flow at each layer or interface under applied external bias.^38^ We note that such electric field profiles do not directly represent the internal built-in field of the device. We further note that the MHP devices show multiple bias-induced electric field peaks (e.g., at the electron/hole transport layers, ETL and HTL). For a given device cross-section, these field peaks are interdependent, and their intensities must be considered relative to each other (i.e., they relate to the fraction of potential drop relative to the total externally applied voltage). This also implies that the individual magnitudes of each electric field peak cannot be directly compared across different devices; rather, the analysis of such KPFM data should compare the total line shape across different devices. Moreover, since KPFM is sensitive to local variations, we performed measurements at multiple locations on the same device and across different devices aged under identical conditions to ensure the consistency of our conclusions derived from KPFM analysis.

The electric field profiles for unaged devices (Figure 4a) show a dominant electric field peak at the ETL side, a screened field in the bulk, and a small field peak at the HTL side. This result is consistent with the expected ion accumulation profiles in the dark condition.^39^ In short, assuming that halide vacancies (cations) are the main mobile ion species and charge neutrality is maintained with a fixed lattice of anions, we expect a wider region of ionic charge depletion near the ETL. This occurs because the built-in field would induce positive halide vacancy motion toward the HTL, where they can accumulate in high density, while the fixed lattice of anions would occupy more space.^39^ The electric field in the region containing a larger width of ionic charge accumulation near the ETL would be more easily measurable within the ∼30 nm resolution of the KPFM instrument compared to the field from the narrow accumulation region near the HTL.

Figure 4b shows the electric field profiles after the device is aged with the metal electrode. We observe a relative decrease in the electric field peak at the ETL side and an increase at the HTL side. This result suggests a greater voltage drop and increase in the equivalent resistance at the perovskite/HTL interface compared with the ETL/perovskite interface. A greater voltage drop at the HTL side may be caused by several possibilities, including a change in the band offset caused by the materials degradation that hinders charge injection or greater electrical transport resistance from the formation of an insulating layer. However, the apparent increase in the field peak at the HTL side may also be impacted by a relative decrease in equivalent resistance at the ETL side.

Indeed, the data suggest substantially lower equivalent resistance at the ETL side due to diffusion of Ag, causing shunting originating at the ETL interface. Relatively high current is measured at −0.5 V in the dark during the KPFM measurement, indicating that the device is more shunted than the fresh devices. The reverse bias KPFM profiles also show minimal voltage drop at both the HTL and ETL, suggesting that the interfaces easily conduct current. Such shunting behavior of the devices during reverse-bias KPFM is not observed under the fresh condition, indicating that aged devices with Ag contacts are more unstable under reverse bias than fresh ones. These reverse bias data suggest that although the increase in the peak at the HTL side could have contributions from the resistive mechanisms noted above; the relative increase in this field peak is largely driven by a relative decrease in voltage drop across the ETL side. The lower voltage drop and shunting that originates at the ETL side are consistent with metal migration through the ETL as observed in SEM and EDS.

Figure 4c shows the cross-sectional electric field profiles from the device that was aged as a partial stack without metal contacts, measured after the fresh contacts were applied. In this case, the electric field profile at the ETL side is maintained, and the field peak at the HTL side increases to a smaller extent. We also note that the reverse bias electric field profiles show more consistent electric field peaks compared to the case aged with metal, and the reverse bias current remained low. These results suggest that shunting is not the primary degradation pathway for devices aged without metal, and the electric field profiles are dominated by a relative increase in resistive degradation modes at both the ETL and HTL. The JV data in Figure 1c clearly show an injection barrier formed from metal corrosion at the ETL side, which would cause an increase in equivalent resistance at the ETL. However, our observation of relative increases at the HTL side suggests that resistive changes must have also occurred at this interface due to changes in energetic offsets and/or resistive material generated at the HTL interface. A greater extent of TCO/HTL degradation, causing electrical resistance, is consistent with the observations of In/Sn movement and voids at this interface, as observed in the SEM/EDS characterization.

We note that the relative changes in KPFM profiles observed here during dark storage exhibit the same trends as similar devices aged under light cycling accelerated stress test conditions,^38,40^ demonstrating the context and importance of these degradation pathways during device operation.

### 3.4 Mitigation Strategy

Figures 5 and 6 highlight possible mitigation strategies for such dark storage degradation of perovskite solar cells. To prevent the diffusion of Ag into the active layer, we replaced the BCP buffer layer with SnO_*x*_. Recently Witteck et al. reported that SnO_*x*_ effectively blocks Ag diffusion into the perovskite active layer during vacuum lamination processing.^41^ Another study by Penukula et al. also demonstrated mitigation of Ag diffusion using a SnO_*x*_ layer.^42^ To achieve better stability during dark storage, we also replaced ITO with FTO as FTO is less reactive compared to ITO.^43,44^

**Figure 5:**
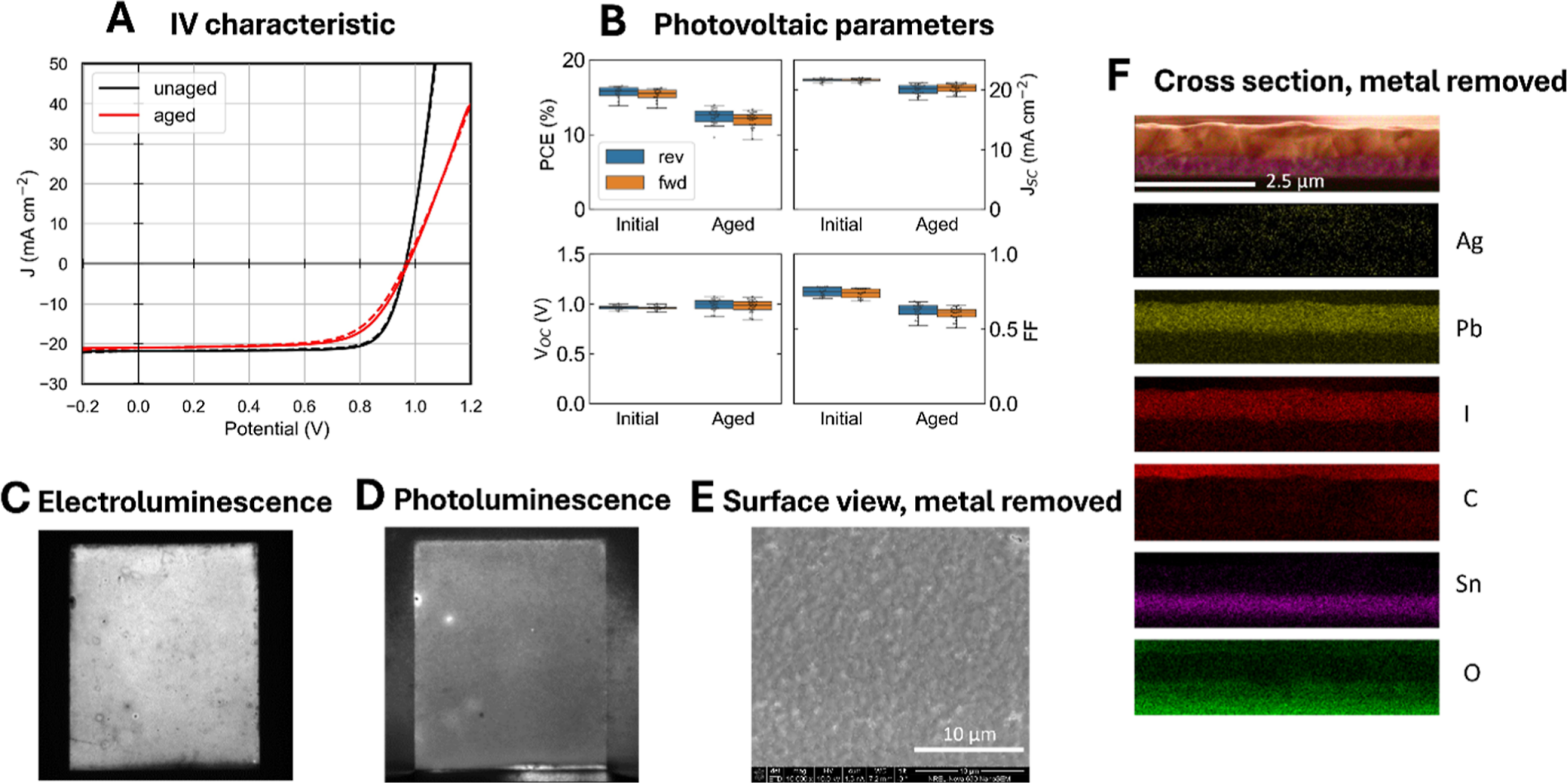
(a) *I*–*V* characteristics, (b) photovoltaic parameters, (c) PL and (d) El images, (e) SEM top view, and (f) EDS maps cross-section of devices on FTO with SnO_*x*_ buffer layer after one year of dark storage.

**Figure 6:**
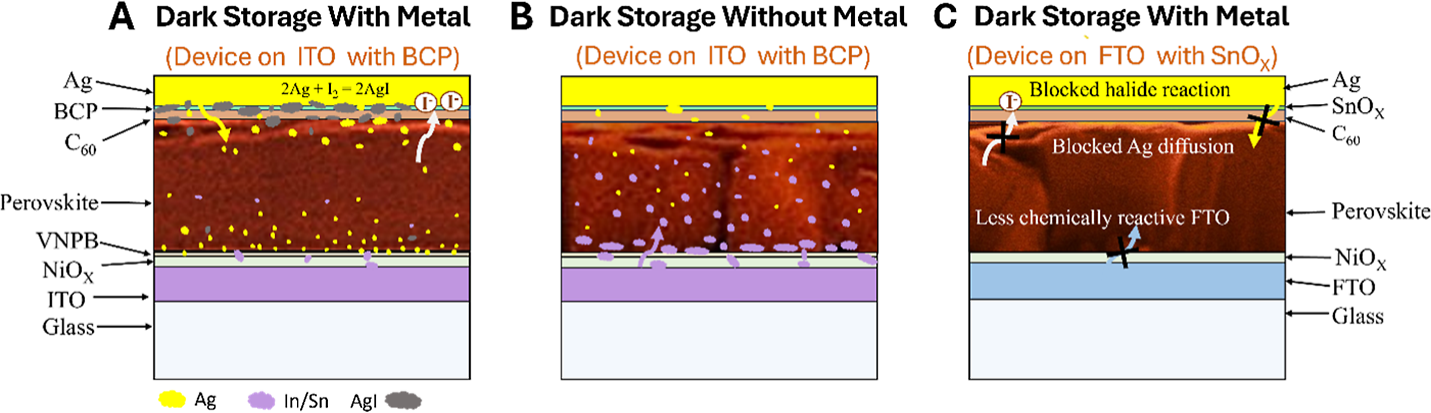
Schematic of degradation mechanisms under dark storage conditions.

Figure 5a,b shows the JV characteristics of the full device stack in fresh conditions and after 1 year of dark storage, with the layer stack of FTO/NiO_*x*_/MHP/C_60_/SnO_*x*_/Ag. We observe improved stability for all JV metrics compared to the devices with a BCP buffer layer and the ITO electrode. The small extent of degradation is mainly attributed to increased series resistance, which causes a lower FF. The small decrease in *J*_SC_ also suggests a slight degradation in charge extraction efficiency. However, *V*_OC_ remains unchanged, indicating that the perovskite absorber is largely stable and that the extent of nonradiative recombination has not significantly increased. The uniform EL intensity (Figure 5c) supports the observation of only minimal electrode degradation. The uniform PL intensity (Figure 5d) further supports that metal-induced absorber degradation has also been largely mitigated.

Figure 5e,f shows the microscopic properties of a device cross-section that was aged in dark storage for one year. Figure 5e shows the surface of the aged devices after the Ag metal electrode was removed with scotch tape. We observed a clear surface without Ag clusters, suggesting mitigation of Ag interfacial reaction and diffusion into the perovskite absorber. In the SEM images of the device cross-section (Figure S2), acquired after the removal of the Ag electrode, the perovskite domain boundaries remain clearly detectable, indicating no significant change in the perovskite absorber.

Figure 5f shows the cross-sectional EDS profiles for the aged devices after Ag electrode removal with scotch tape. We do not observe a significant Ag signal in the perovskite absorber. Such a result suggests that the SnO_*x*_ buffer layer effectively blocks Ag diffusion and suppresses the metal-induced degradation of the perovskite absorber. The SnO*_x_* layer also blocks halide reactions with the Ag contact, mitigating reaction pathways that would have formed AgI. Also, these devices show an intense Sn signal near the FTO contact without extending into the MHP, and no voids at the FTO contact. This observation suggests that Sn does not migrate from FTO into the perovskite layer, as observed with ITO, likely due to the chemically inert nature and superior thermal stability of FTO compared to ITO. Our results suggest that the SnO_*x*_ buffer layer and FTO electrode together provide a better stability of MHP devices under dark storage with increased shelf life by mitigating Ag diffusion from the back electrode as well as suppressing electrochemical reactions at the transparent conductive oxide.

Figure 6 schematically illustrates the overall degradation pathways under a dark inert atmosphere and the possible mitigation strategy. Figure 6a represents the degradation pathway of the full device stack, where the dominant degradation pathway includes Ag diffusion and accumulation near the ITO interface, along with halide reactions with Ag. This causes the formation of AgI and changes in the perovskite morphology, leading to a smooth absorber layer with no detectable domain boundaries. The device stored without metal (Figure 6b) showed a different degradation pathway dominated by corrosion of ITO that causes the formation of voids near the ITO interface and diffusion of Sn into the device stack. In Figure 6c, we present the mitigation strategy of these degradation pathways. The SnO*_x_* buffer layer effectively blocks Ag migration and metal-halide reactions. Replacement of ITO by less reactive FTO suppresses the electrochemical reactions at the transparent electrode and mitigates the diffusion of Sn and In.

## 4 Conclusions

We investigated degradation mechanisms in p-i-n perovskite solar cells that were stored in the dark in an argon atmosphere for approximately 1 year. We compared full devices that were stored with the Ag metal contact to devices that were stored as a partial stack with all layers excluding the Ag metal contact. We found that Ag diffusion was the major source of degradation for devices stored with the metal contact, causing shunting that originates at the ETL, Ag accumulation at the HTL side, and loss of the absorber domain structure. This resulted in external-voltage-induced electric field profiles dominated by the voltage drop at the HTL side with a minimal signal at the ETL side. The devices stored without metal contact showed a different degradation pathway. With fresh Ag applied, the devices showed some *J*_SC_ loss, but overall, much better performance than the devices stored with Ag. However, a short additional period of dark storage resulted in Ag corrosion and the development of an injection barrier, demonstrating instability of the perovskite absorber, which created degradation products that rapidly corrode the metal electrode. The device stored without metal showed degradation primarily by corrosion reactions at the ITO contact, resulting in voids and Sn/In diffusion. Importantly, the KPFM results reveal distinct electric field profiles depending on storage conditions with or without Ag. This shows that degradation at the ITO interface leads to different resistive losses when Ag diffusion is absent, highlighting an independent ITO degradation pathway that can occur if metal diffusion is mitigated. We demonstrated that these degradation pathways could be mitigated by changes to the device stack, including a SnO_*x*_ blocking layer at the ETL side and replacing ITO with FTO at the HTL side. Taken together, this study provides a useful demonstration of degradation pathways under dark storage to inform further improvement of the perovskite device stability. Furthermore, our multimodal characterization approach highlights KPFM as a useful method to link changes in interface composition observed with electron microscopy to the electrical characteristics of the interfaces.

## Supplementary Material



## Notes

XRD scans of the unaged perovskite control sample, bare ITO substrate, and aged devices and cross-sectional SEM images from three different areas of devices on FTO with the SnO_*x*_ buffer layer after one year of dark storage (PDF)
