# Supplementary material for: Multiscale Characterization of Electrode-Induced Degradation in Perovskite Solar Cells
Source: ACS Appl Energy Mater. 2026 Feb 16;9(5):2503–12. doi: 10.1021/acsaem.5c03347 (PMC12977041; doi:10.1021/acsaem.5c03347)
Supplement: Supplementary file 1 [file ae5c03347_si_001.pdf]

Supporting information

# Multiscale characterization of electrode-induced degradation in perovskite solar cells

*Goutam Paul, Jackson W. Schall, Harvey L. Guthrey, Marc Migliozzi, Robert Tirawat, Dennice M. Roberts, Steven W. Johnston, Mowafak M. Al-Jassim, Chun-Sheng Jiang, Axel F. Palmstrom, Dana B. Kern**

National Renewable Energy Laboratory, 15013 Denver West Parkway, Golden, CO 8040, United States.

*Corresponding author. E-mail: Dana.Kern@nrel.gov

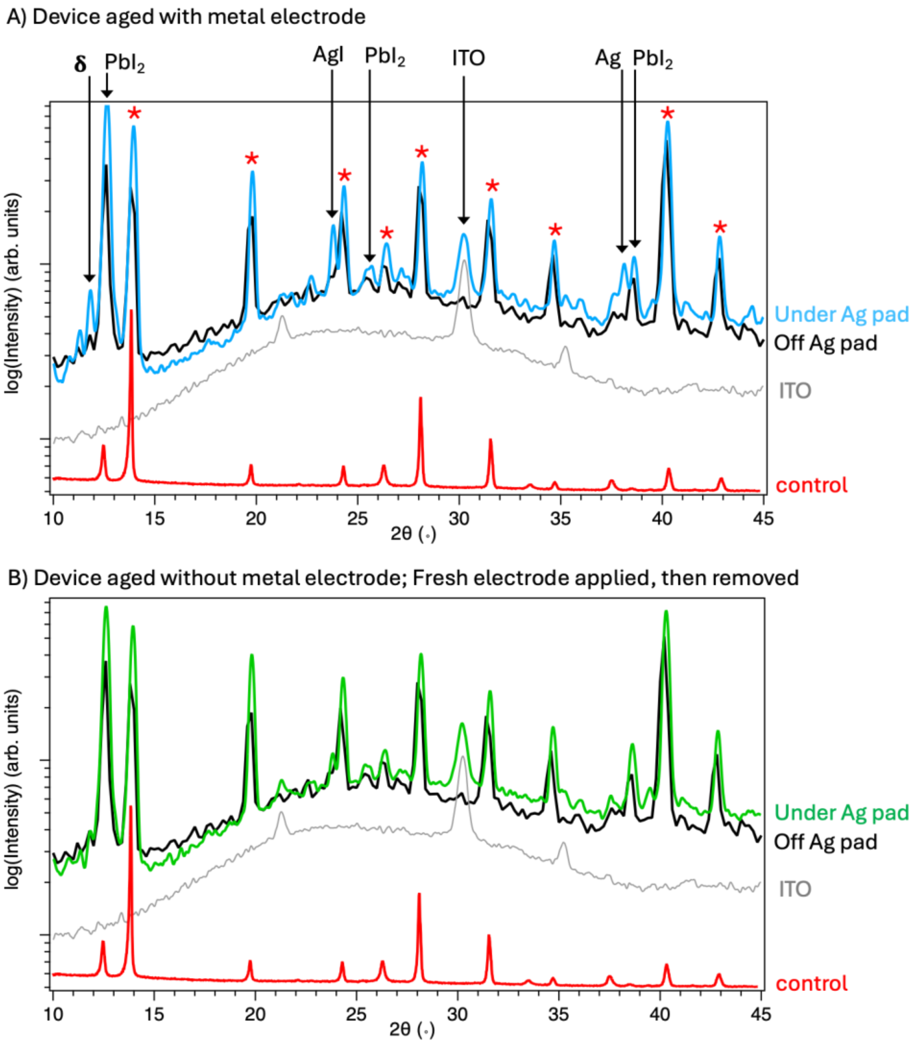


**Fig. S1:** X-ray diffraction spectra of control and degraded device.

X-ray diffraction (XRD) scans are collected using a Rigaku Smartlab diffractometer with Cu–Kα radiation and parallel beam optics. Data collected with a 0.5 mm incident slit and a 0.25 deg/min integration time. The XRD measurements show a comparison of the unaged perovskite control sample in red, bare ITO substrate in gray, and aged devices. The area beneath the Ag pad shows additional peaks that are not observed in the area away from the Ag pad. For the device aged with Ag present, we observe peaks that we attribute to silver iodide (AgI) at 23.8 degrees and silver metal (Ag) at 38.1 degrees.[1-3] This indicates silver metal diffuses into the perovskite layer, and that reactions with the perovskite active layer cause AgI byproducts. The devices aged with metal present also show a peak at 11.8 degrees, which is consistent with studies showing the non-photoactive yellow δ-phase of $FAPbI_3$.[4-5]

We also observe degradation of the perovskite active layer where peaks are present in both devices that were aged with and without Ag present. We note that the device aged without Ag

present had Ag applied, and then subsequently removed for characterization. The peaks at 12.6, 25.5, and 38.6 degrees indicate $PbI_2$ formation.[6][refs] It is possible that the peak at 31.3 degrees is enhanced in the degraded sample due to formation of metallic lead ($Pb^0$).[6-7]

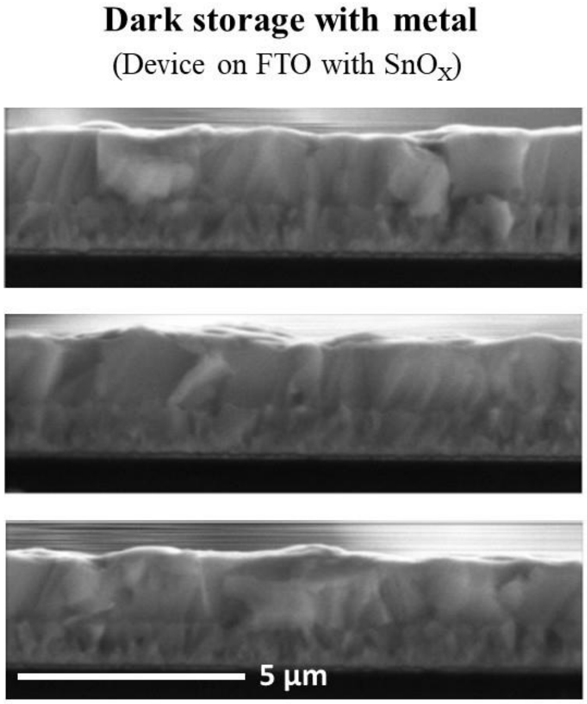


**Fig. S2:** Cross-sectional SEM images from three different areas of devices on FTO with $SnO_x$ buffer layer after one year of dark storage.

**References:**

(1) Han, Y.; Meyer, S.; Dkhissi, Y.; Weber, K.; Pringle, J. M.; Bach, U.; Spiccia, L.; Cheng, Y.-B., Degradation Observations of Encapsulated Planar Ch3nh3pbi3 Perovskite Solar Cells at High Temperatures and Humidity. *Journal of Materials Chemistry A* **2015,** *3* (15), 8139–8147.

(2) Zhang, T.; Meng, X.; Bai, Y.; Xiao, S.; Hu, C.; Yang, Y.; Chen, H.; Yang, S., Profiling the Organic Cation-Dependent Degradation of Organolead Halide Perovskite Solar Cells. *Journal of Materials Chemistry A* **2017,** *5* (3), 1103–1111.

(3) Kato, Y.; Ono, L. K.; Lee, M. V.; Wang, S.; Raga, S. R.; Qi, Y., Silver Iodide Formation in Methyl Ammonium Lead Iodide Perovskite Solar Cells with Silver Top Electrodes. *Advanced Materials Interfaces* **2015,** *2* (13), 1500195.

(4) Elsayed, M. R. A.; Elseman, A. M.; Abdelmageed, A. A.; Hashem, H. M.; Hassen, A., Synthesis and Numerical Simulation of Formamidinium-Based Perovskite Solar Cells: A Predictable Device Performance at Nis-Egypt. *Scientific Reports* **2023,** *13* (1), 10115.

(5) Xie, L.-Q.; Chen, L.; Nan, Z.-A.; Lin, H.-X.; Wang, T.; Zhan, D.-P.; Yan, J.-W.; Mao, B.-W.; Tian, Z.-Q., Understanding the Cubic Phase Stabilization and Crystallization Kinetics in Mixed Cations and Halides Perovskite Single Crystals. *Journal of the American Chemical Society* **2017,** *139* (9), 3320–3323.

(6) Kundu, S.; Kelly, T. L., In Situ Studies of the Degradation Mechanisms of Perovskite Solar Cells. *EcoMat* **2020,** *2* (2), e12025.

(7) Liang, J.; Hu, X.; Wang, C.; Liang, C.; Chen, C.; Xiao, M.; Li, J.; Tao, C.; Xing, G.; Yu, R.; Ke, W.; Fang, G., Origins and Influences of Metallic Lead in Perovskite Solar Cells. *Joule* **2022,** *6* (4), 816–833.
